# Crystal structure and analytical profile of 1,2-di­phenyl-2-pyrrolidin-1-yl­ethanone hydro­chloride or ‘α-D2PV’: a synthetic cathinone seized by law enforcement, along with its diluent sugar, myo-inositol

**DOI:** 10.1107/S2053229624000561

**Published:** 2024-01-22

**Authors:** Matthew R. Wood, Ivan Bernal, Roger A. Lalancette

**Affiliations:** a Carl A. Olson Memorial Laboratories, Department of Chemistry, Rutgers University, 73 Warren St., Newark, NJ 07102, USA; b Ocean County Sheriff’s Office, Forensic Science Laboratory, Toms River, NJ 08753, USA; c Molecular Sciences Institute, School of Chemistry, University of the Witwatersrand, Private Bag 3, 2050 Johannesburg, South Africa; Rigaku Americas Corporation, USA

**Keywords:** crystal structure, cathinones, bath salts, racemic drugs, sugars, inositol, asymmetric units, mol­ecular overlays, novel psychoactive substances, π–π inter­actions, hydrogen bonding

## Abstract

A confiscated package of street drugs was characterized by the usual mass spectral (MS) and FT–IR analyses. The confiscated powder material was highly crystalline and was found to consist of two very different species, accidentally of sizes convenient for X-ray diffraction. Thus, one each was selected and redundant com­plete sets of data were collected at 100 K using Cu *K*α radiation. The selected crystals contained: (*a*) 1,2-diphenyl-2-(pyrrolidin-1-yl)ethanone hy­dro­chloride hemihydrate or 1-(2-oxo-1,2-di­phenyl­eth­yl)pyrrolidin-1-ium chloride hemihydrate, C_18_H_20_NO^+^·Cl^−^·0.5H_2_O, (I), a synthetic cathinone called ‘α-D2PV’, and (*b*) the sugar myo-inositol, C_6_H_12_O_6_, (II), probably the only instance in which the drug and its diluent have been fully characterized from a single confiscated sample. Moreover, the structural details of both are rather attractive showing: (i) inter­esting hydrogen bonding observed in pairwise inter­actions by the drug mol­ecules, mediated by the chloride counter-anions and the waters of crystallization, and (ii) π–π inter­actions in the case of the phenyl rings of the drug which are of two different types, namely, π–π stacking and edge-to-π. Finally, the inositol crystallizes with *Z*′ = 2 and the resulting diastereoisomers were examined by overlay techniques.

## Abstract 2

A confiscated package of street drugs was highly crystalline and was found to consist of two very different species accidentally of sizes convenient for X-ray diffraction, namely, 1,2-diphenyl-2-(pyrrolidin-1-yl)ethanone hydro­chloride or ‘α-D2PV’ and the sugar myo-inositol.

## Introduction: useful historical notes and commentaries

In 2021, as part of a law enforcement investigation, an off-white crystalline powder was submitted for analysis. This submission contained two com­ponents: α-pyrrolidino-2-phenyl­aceto­phenone (called α-D2PV), which is an *N*-pyr­roli­dinyl substituent of natural cathinone, and myo-inositol, a common sugar. The sample was initially identified using GC–MS and the structures of both materials were confirmed by single-crystal X-ray diffraction.

α-D2PV, (I), belongs to a class of stimulants, ‘synthetic cathinones’, that are simple modifications of the chemical structure of cathinone. Cathinone is a naturally occurring chemical found in the khat plant (*Catha edulis*), commonly grown and used in East Africa (Kalix, 1992 ▸). Cathinone and several derivatives have been scheduled by the DEA as controlled substances, leading to the syntheses of new synthetic cathinone com­pounds (often referred to as ‘bath salts’) which were developed to produce similar psychotropic and stimulant effects as ‘legal highs’ (Zawilska & Wojcieszak, 2013 ▸), and to circumvent the ‘controlled substances’ list. In this instance, α-D2PV is obtained by the substitution of a pyrrole ring in place of the amine group and a phenyl group on the α-C atom (Scheme 1). 
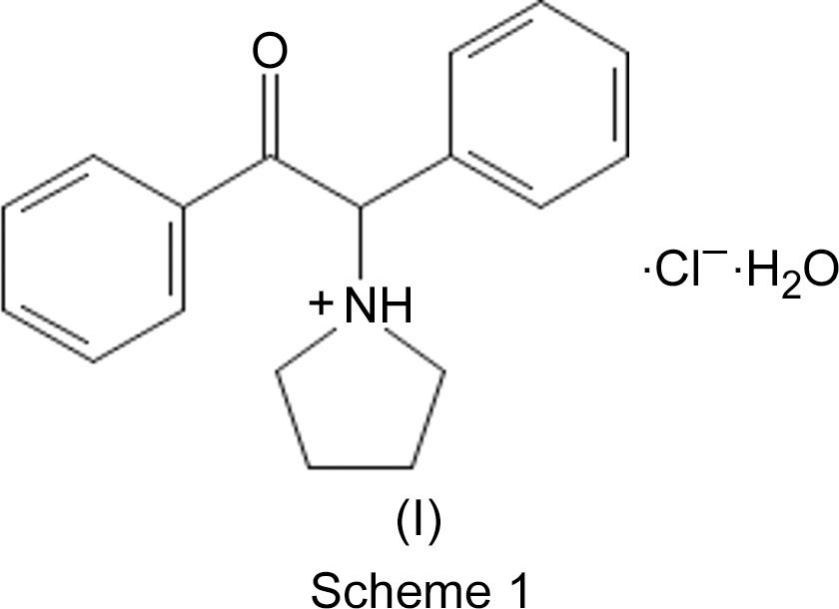


Very little pharmacological information is available beyond reports on recreational drug-use websites. Due to the fast development of new designer drugs, we find it important to provide analytical data on as many new addictive com­pound(s) as become available to assist in law enforcement and toxicological investigations, which serve as an addition to a growing list of new psychotropic com­pounds; researchers at the University of Silesia have been adding to this list by making recent contributions (Rojkiewicz *et al.*, 2020 ▸; Kuś *et al.*, 2017 ▸, 2019 ▸).

Inositol has an inter­esting history as a natural product because it is produced by many plants, such as citrus, beans, corn, sesame, *etc.*, and from glucose by the human body. Inter­estingly, the substance is not optically active because of the symmetrical hy­droxy­lation of the six aliphatic C atoms of the cyclo­hexane central ring. For an inter­esting description of its history and early crystallographic background, we recommend the papers by Rabinovich & Kraut (1964 ▸) [Cambridge Structural Database (CSD; Groom *et al.*, 2016 ▸) refcode: MYINOL] and Rebecca *et al.* (2012 ▸) (MYINOL02). Scheme 2 shows myo-inositol, (II). 
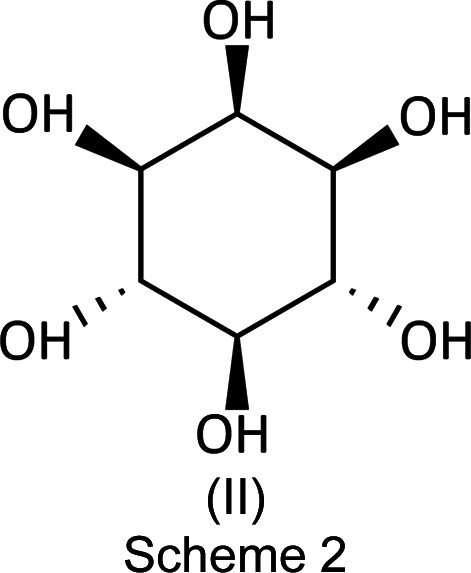


## Experimental (methods and materials)

### Sample preparation

The com­pound of inter­est was initially received as part of a law enforcement investigation of suspected controlled dan­gerous substances. A portion of approximately 5 mg was dis­solved in 1 ml of LC–MS grade methanol supplied by Fisher Chemical (Palo Alto, CA, USA) for GC–EI–MS (gas chromatography–electron ionization–mass spectrometry) analysis. A small portion of the sample material was ground and analyzed by ATR–FT–IR (attenuated total reflectance–Fourier transform–infrared spectroscopy) without further sample preparation. A separate portion of the drug material was examined microscopically and two suitable single crystals were selected without further preparation for the X-ray diffraction experiment. Both crystals had distinctly different morphologies: the one that would prove to be the title synthetic cathinone, (I), was a colourless prism; the second, a common illicit drug diluent, the sugar inositol, (II), was a colourless parallelepiped. Additionally, a reference standard of the drug com­pound was purchased from Cayman Chemical (Ann Arbor, Michigan, USA) for com­parison and confirmation.

### Mass spectral analysis of (I)

The mass spectral analysis of (I) were performed using GC–EI–MS on the law-enforcement-seized sample.

**Figure 1:**
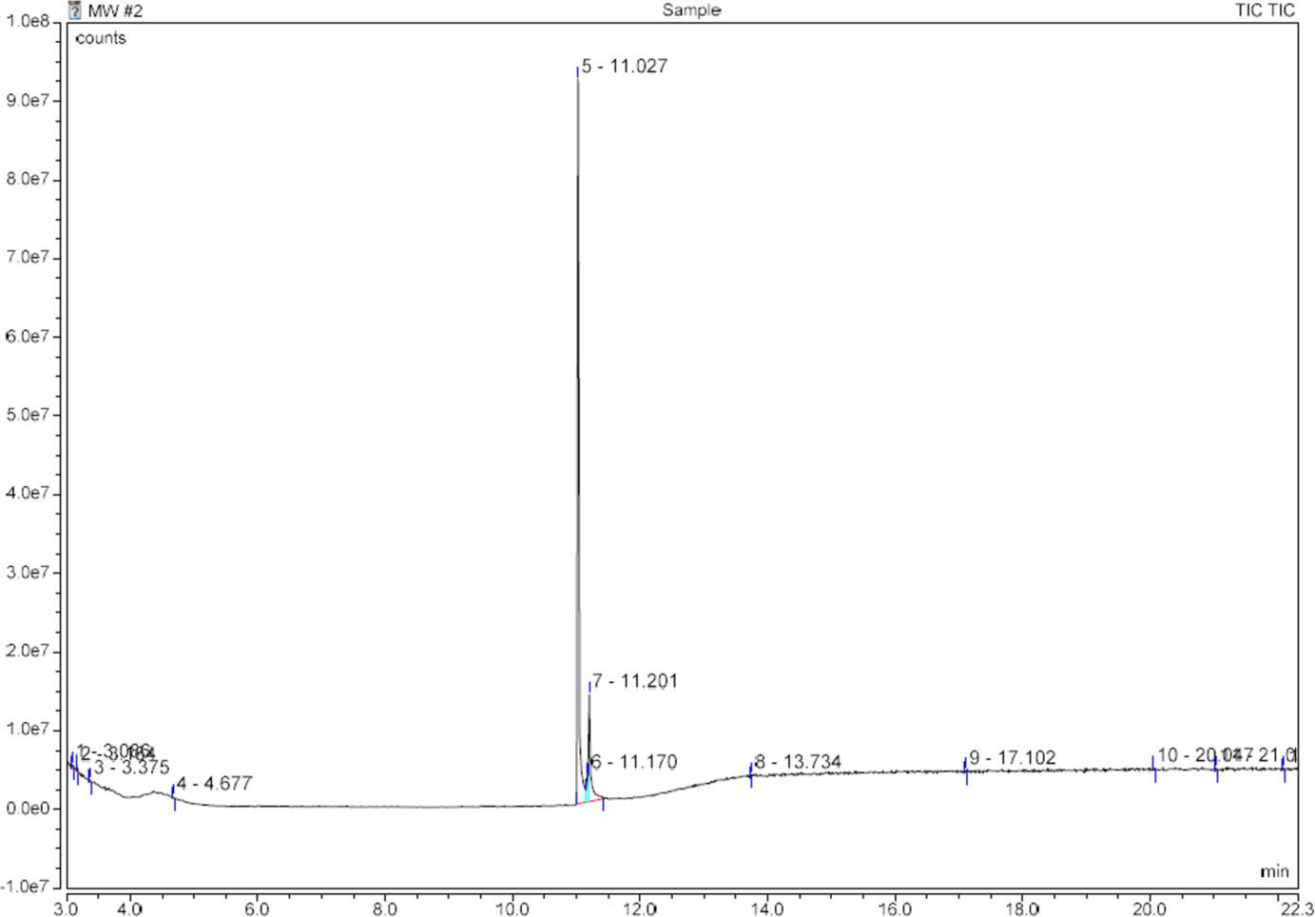
GC spectrum of α-D2PV, (I). The main sample peak at 11.03 min represents the synthetic cathinone α-D2PV. The small peaks at 11.20 min represent the thermal degradation of α-D2PV in the GC injection port (see *Discussion* section).

**Figure 2:**
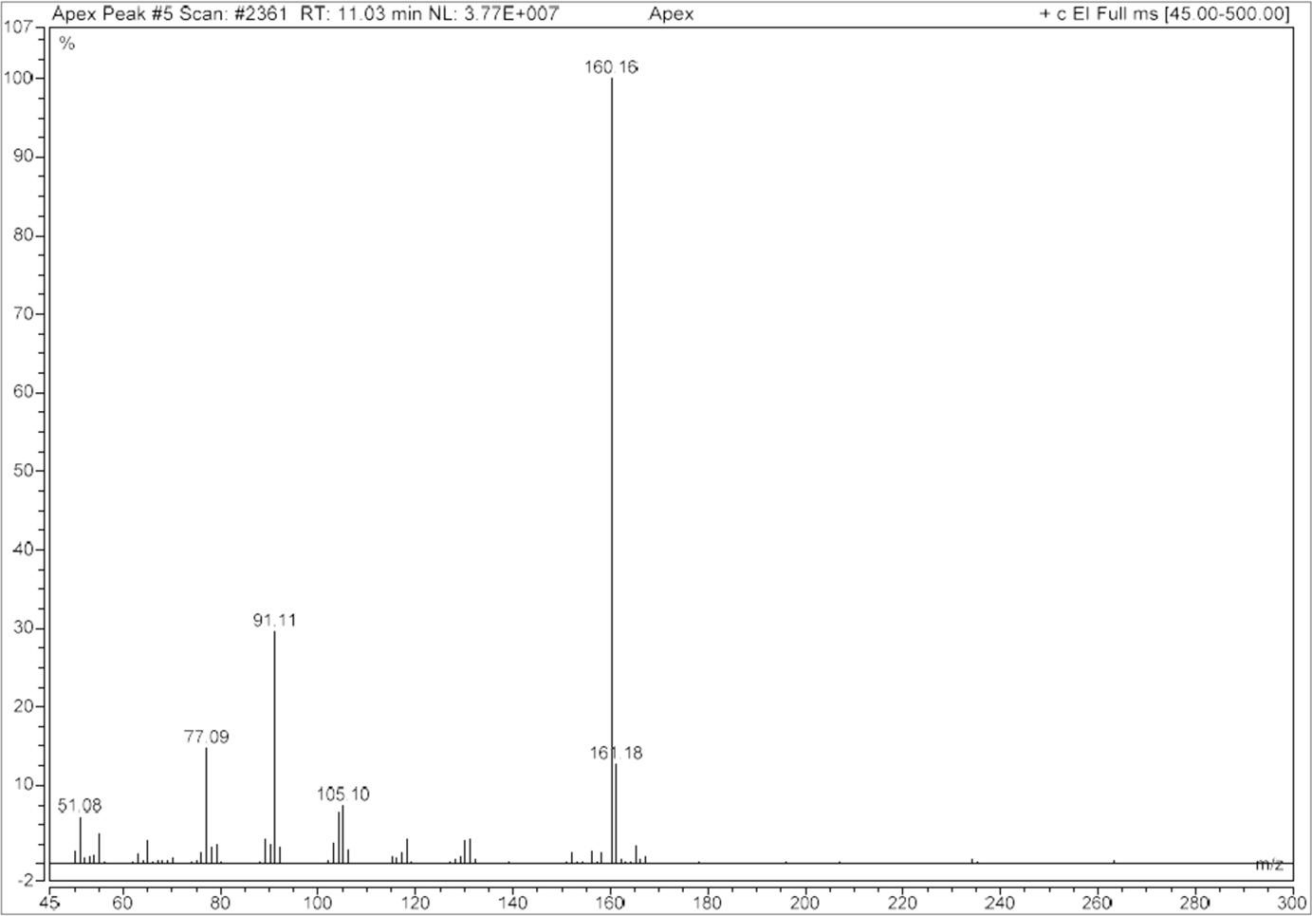
Mass spectral fragmentation of α-D2PV, (I).

A Thermo Scientific Trace 1310 Gas Chromatograph ISQ 7000 Mass Spectrometer [single quadrupole GC–EI–MS, utilizing a Restek Rtx-5MS (5% diphen­yl–95% methyl cross-bonded polysiloxane) 30 m × 0.25 mm ID × 0.25 µm film column (catalogue No. 12623)] was used as part of the general drug analytical scheme of the forensic laboratory. The GC and MS parameters can be found in Table 1 ▸ and the GC spectrum of α-D2PV, (I), is shown in Fig. 1 ▸. The mass spectral fragmentation pattern for α-D2PV, (I), is shown in Fig. 2 ▸.

**Figure 3:**
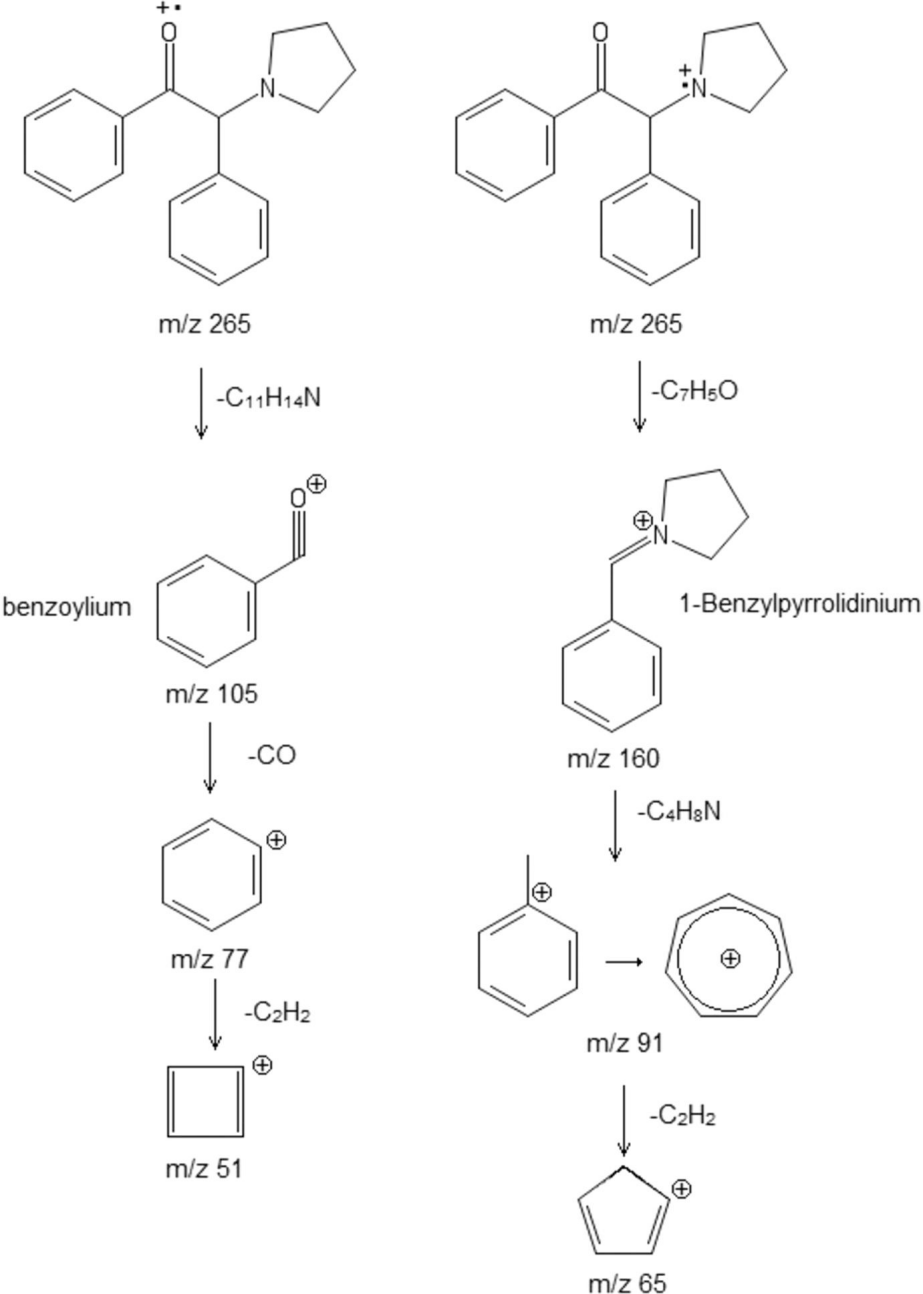
The proposed major mass spectra fragmentation ions of α-D2PV, (I).

Cathinone fragmentation patterns are dominated by α-cleavage at both the amine and the carbonyl groups. The ions produced by the EI fragmentation of α-D2PV are consistent with previously described EI–MS fragmentation of α-pyrrolidino­phenone synthetic cathinones (Zuba, 2012 ▸; Matsuta *et al.*, 2014 ▸; Qian *et al.*, 2017 ▸; Davidson *et al.*, 2020 ▸). In the case of α-D2PV, the fragmentation produced the expected base 1-benzyl­idenepyrrolidinium ion at *m*/*z* 160 and the benzoyl­ium ion at *m*/*z* 105 (Fig. 2 ▸). The proposed fragmentation mechanism (Fig. 3 ▸) is based on the extensive work of Davidson *et al.* (2020 ▸). Subsequent fragmentation of the 1-benzyl­idenepyrrolidinium ion yields a tropylium ion at 91 *m*/*z* and the cyclo­penta-1,3-­diene-1-ylium ion at 65 *m*/*z*; subsequent fragmentation of the benzoyl­ium produces a phenyl­ium ion at 77 *m*/*z* and the cyclo­butadien-4-ylium ion at 51 *m*/*z*. Fig. 3 ▸ shows a proposed fragmentation mechanism of α-D2PV, (I), including the possible ions involved.

### Direct analysis of the seized drug material (I) by ATR–FT–IR

**Figure 4:**
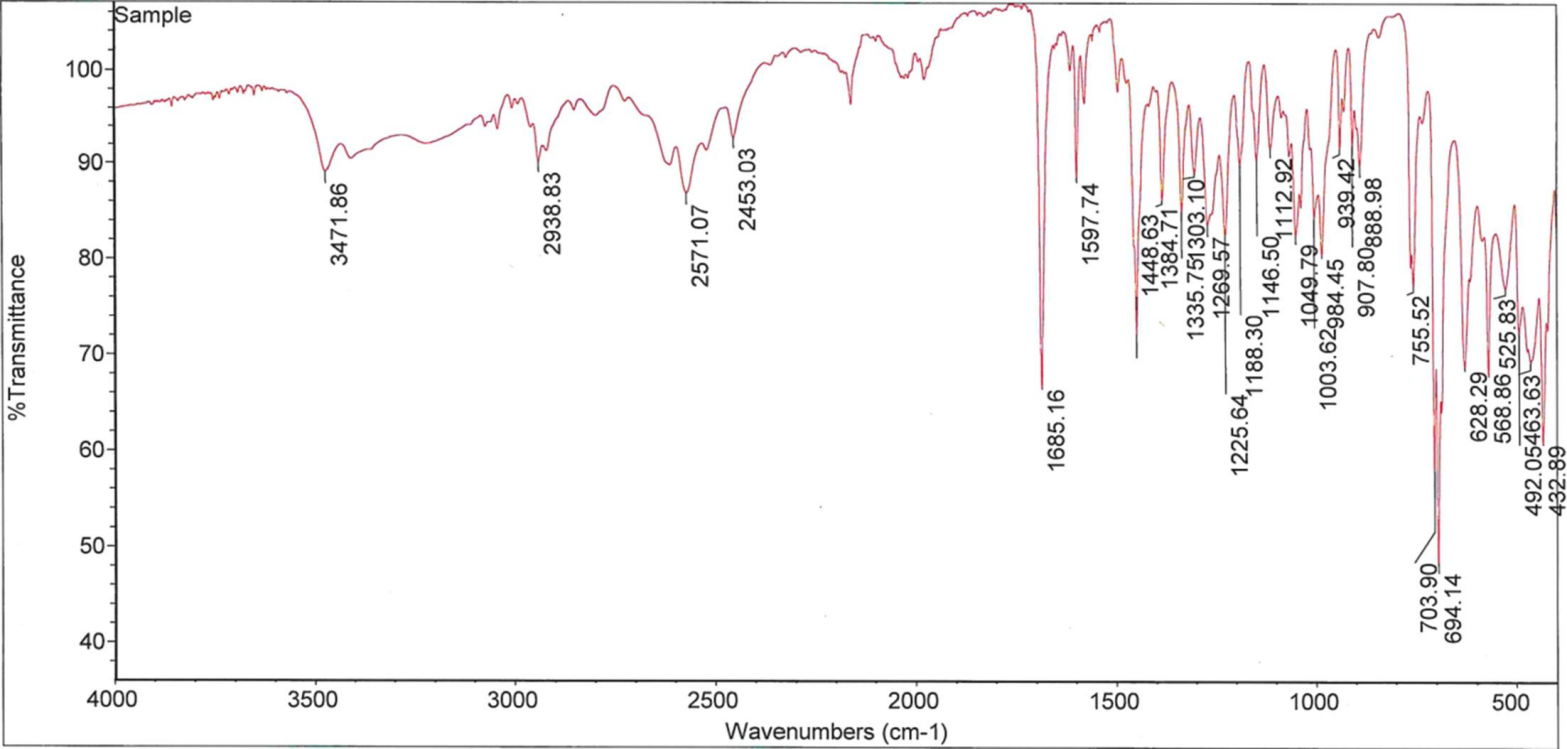
FT–IR spectrum of α-D2PV, (I).

The IR spectrum (Fig. 4 ▸) was obtained with a Nicolet iS50 FT–IR spectrometer (Thermo Scientific), using attenuated total reflectance (ATR), and the spectrum was collected in the wavenumber range 4000–400 cm^−1^.

### X-ray data collection, structure solutions, and refinements of α-D2PV, (I), and inositol, (II)

Crystals of (I) and (II) were secured to a micromount fiber loop using Paratone-N oil. The crystal dimensions, as well as the pertinent crystal information for both com­pounds, are given in Table 2 ▸. The SCXRD data for both materials were collected at 100 K on a Rigaku XtaLAB Synergy-S Dual Source diffrac­tometer with a PhotonJet Cu-microfocus source (λ = 1.54178 Å) and a HyPix-6000HE hybrid photon counting (HPC) detector. To ensure com­pleteness and desired redundancy, data collection strategies were calculated using *CrysAlis PRO* (Rigaku OD, 2022 ▸). Subsequent data processing was also performed in *CrysAlis PRO*. Using the SCALE3 ABSPACK scaling algorithm (Rigaku OD, 2022 ▸), empirical and numerical (Gaussian) absorption corrections were applied to the data (faces were determined using face-indexing in *CrysAlis PRO*). The structures were solved *via* intrinsic phasing methods using *SHELXT* in *OLEX2* (Dolomanov *et al.*, 2009 ▸) and refined by full-matrix least-squares techniques against *F* ^2^ (*SHELXL*; Sheldrick, 2015*a* ▸), first in the *OLEX2* (Dolomanov *et al.*, 2009 ▸) graphical user inter­face, and later using *SHELXTL* (Sheldrick, 2015*b* ▸).

All H atoms were placed either according to their electron-density Q-peaks or were attached *via* the riding model in idealized positions. Data for both (I) and (II) are given in Table 2 ▸. Mol­ecular overlay diagrams were generated using *Mercury* (Macrae *et al.*, 2020 ▸) and *DIAMOND* (Putz & Brandenburg, 2019 ▸).

## Description of the structures

**Figure 5:**
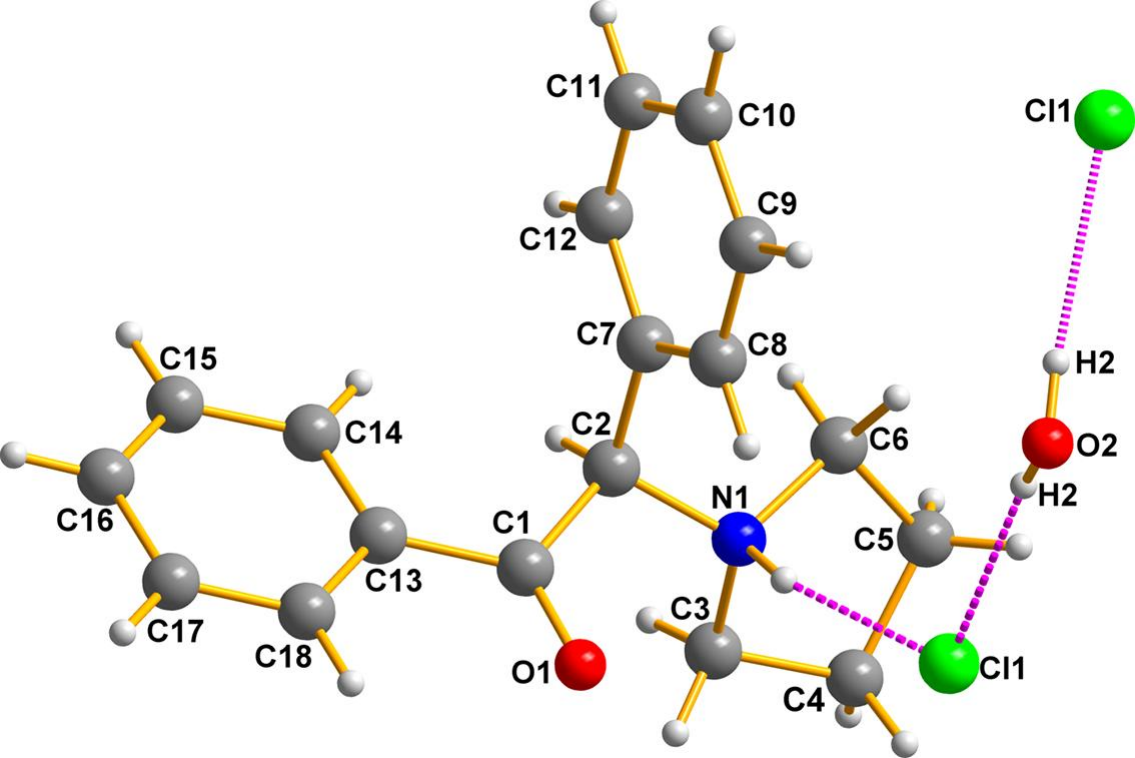
The mol­ecular structure of α-D2PV, (I). The ammonium cations are linked to one another by hydrogen bonds to Cl⋯H_2_O⋯Cl fragments as displayed above. This view is intended primarily to show the stereochemistry of the cationic drug; however, there are additional bonding inter­actions linking the elements of the lattice tightly (see Figs. 5 ▸ and 6 ▸).

**Figure 6:**
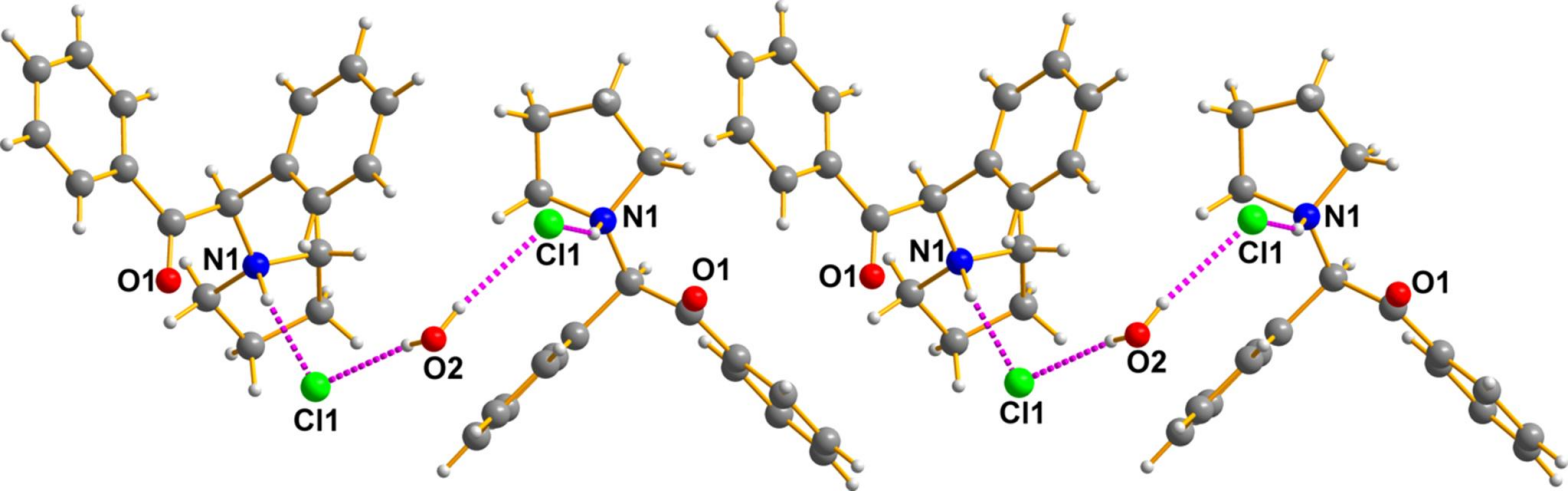
There are layers of cathinone cations above and below what is presented. These are not shown to avoid clutter. Generic atoms labels without symmetry codes have been used.

The seized crystals contain two species (easily isolated under the microscope): a colourless prism was the synthetic cathinone (I) and a colourless parallelepiped was myo-inositol (II). Fig. 5 ▸ shows the cationic amine, the chloride counter-anion, and the water of crystallization all held together by hydrogen bonds. The hydrogen bonds of the chloride anions to the protons of the ammonium N atom are important for the packing, as illustrated in Figs. 6 ▸ and 7 ▸. A mol­ecular diagram for (I) is given in the supporting information.

The overall packing is difficult to display in a single view because of its com­plex three-dimensional character. Fig. 6 ▸ shows additional features of the packing, which is also deceptive because it gives the impression that the cations are only linked exclusively in pairs by the Cl⋯H_2_O⋯Cl fragments. That such is not the case is clearly shown below.

**Figure 7:**
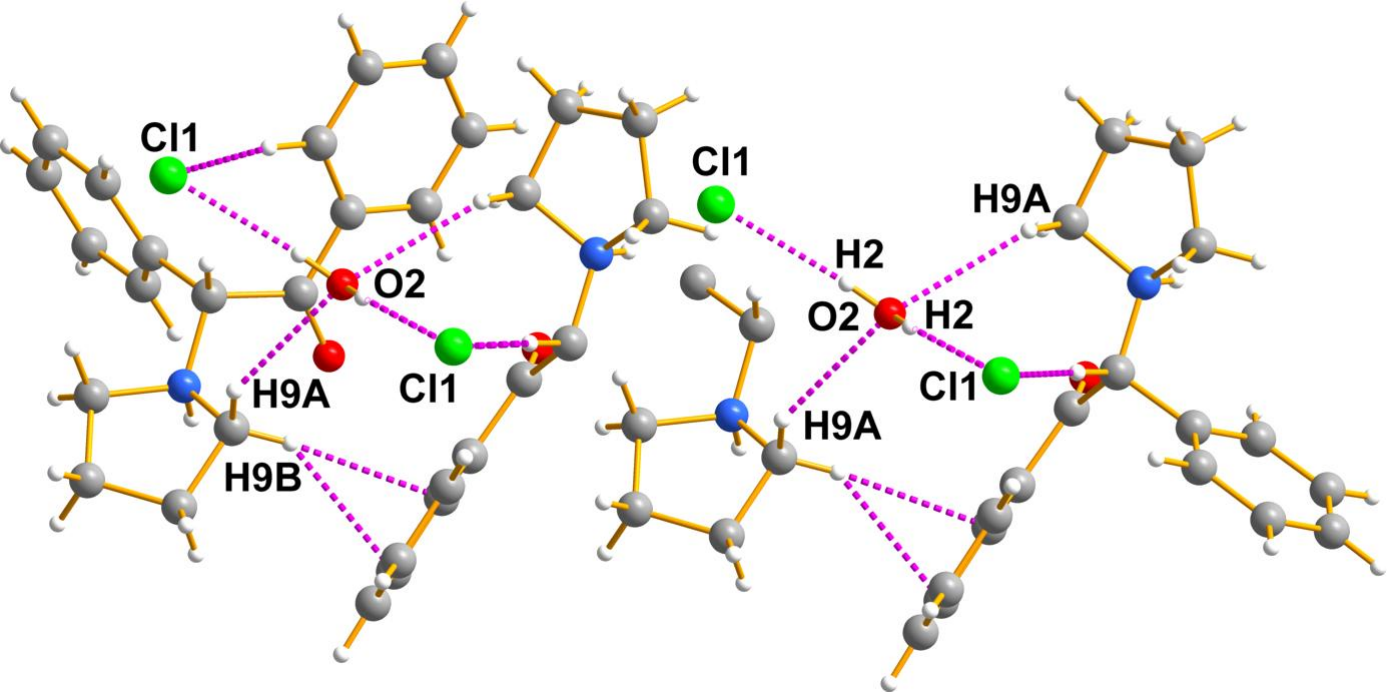
This figure intends to show that the lattice is held together by a multitude of hydrogen bonds of the usual N—H⋯O, O—H⋯O, and Cl⋯H types, but that, in addition, there are large numbers of meaningful hydrogen-bond contacts shorter than 2.9 Å that help stabilize the lattice given their size and numbers. Generic atoms labels without symmetry codes have been used.

Next, Fig. 7 ▸ shows the hydrogen bonding between the quarternary amine group of the drug, the chloride counter-anion, the water of hydration, and then the chloride of the next mol­ecule.

**Figure 8:**
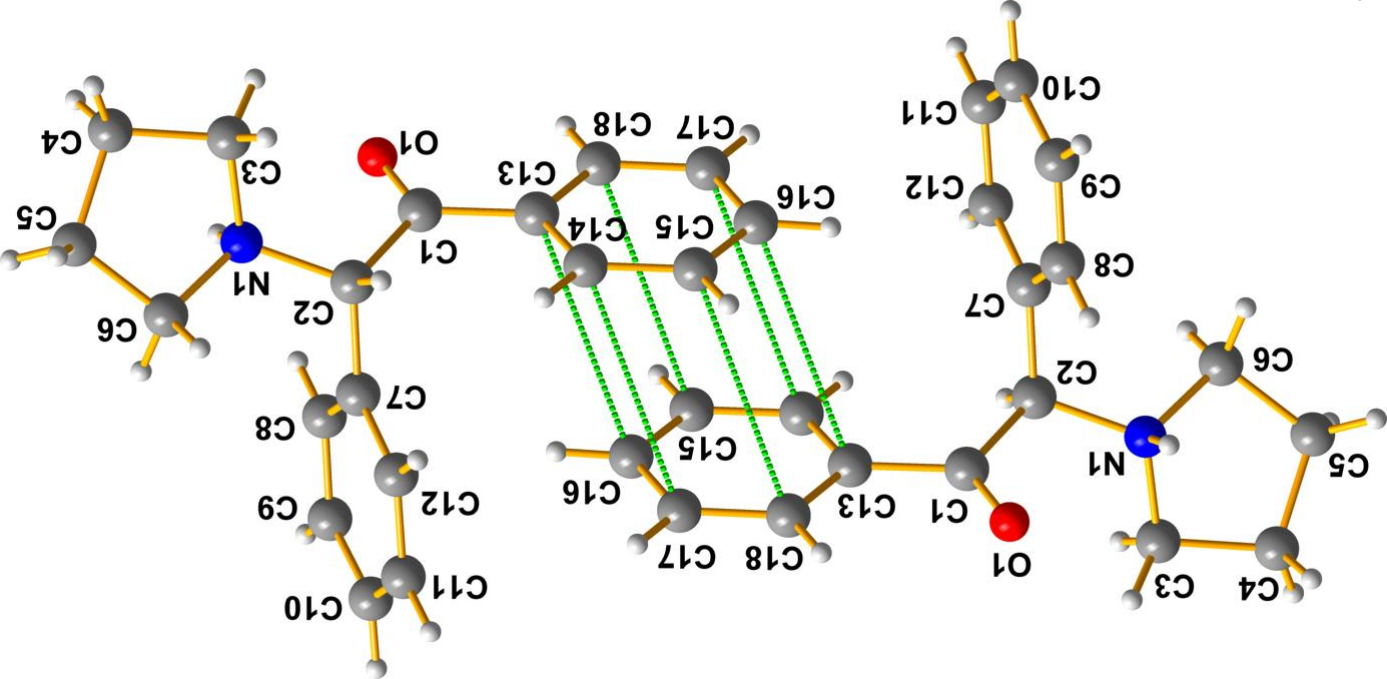
A view of the π–π inter­actions between the arene rings of adjacent cations in (I). Generic atoms labels without symmetry codes have been used.

There are important π–π inter­actions (see Fig. 8 ▸) between the phenyl rings of adjacent cations that cannot simultaneously be displayed in the figures described above.

The π–π inter­actions in this crystal are very substantial; the C—C distances between the rings range from 3.6600 (17) to 3.6985 (17) Å. These belong to the short type as discussed by Janiak (2000 ▸), whose paper gives a critical account on π–π stacking in metal com­plexes with aromatic nitro­gen-con­tain­ing ligands. The ring centroids here are 3.684 Å apart, and the angle between the normal to the planes of the two phenyl rings is 108.9°.

**Figure 9:**
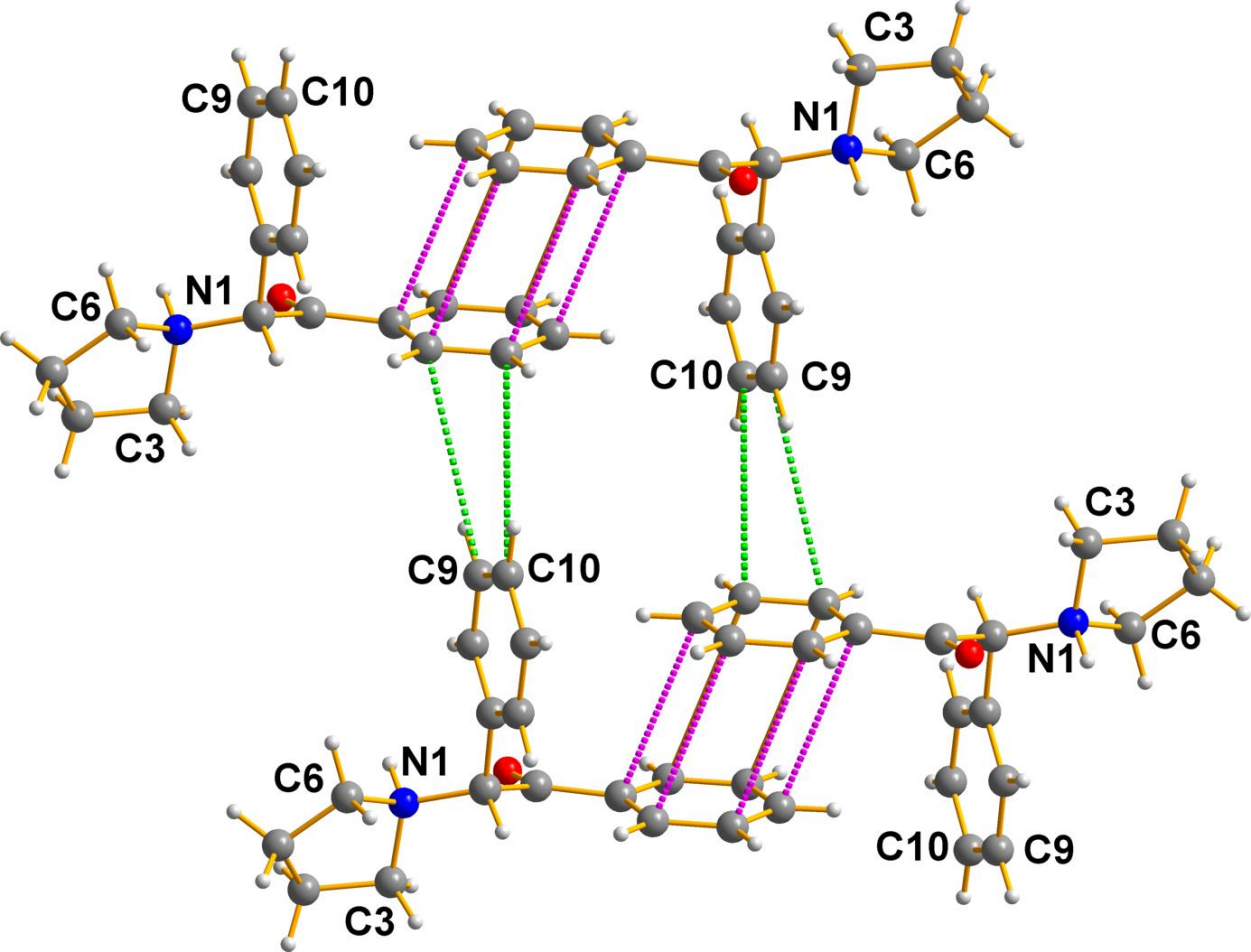
Pairs, similar to those in Fig. 7 ▸, inter­act as shown here. Generic atoms labels without symmetry codes have been used.

It is inter­esting to note that there is another inter­action between the aromatic moieties in this structure, namely, that the cations depicted in Fig. 8 ▸ also contain a face-to-edge contact depicted in Fig. 9 ▸. Note that atoms C9 and C10 (H atoms omitted for clarity) inter­act closely with those of C14 [4.3277 (18) Å] and C15 [4.690 (2) Å] (symmetry code: *x* + 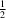 , *y* + 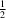 , *z*). Thus, the entire lattice becomes very tightly bound.

### The sugar inositol, (II) – the diluent

**Figure 10:**
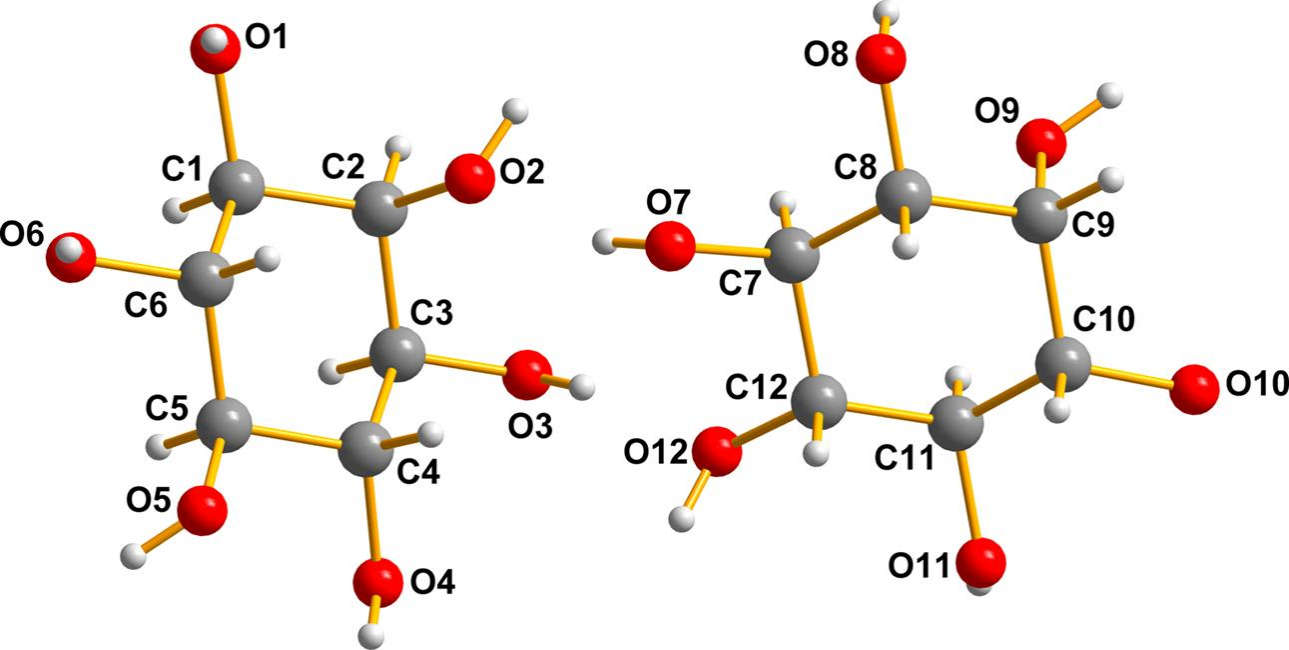
The two mol­ecules of inositol, (II), in the asymmetric unit are shown with the numbering system necessary to describe the overlay diagram shown in Fig. 10 ▸.

The sugar inositol, (II), crystallizes with two mol­ecules in the asymmetric unit (*Z*′ = 2), depicted in Fig. 10 ▸. A mol­ecular diagram for (II) is given in the supporting information.

It is clear that the pair are related by a ‘near inversion’ noncrystallographic center located between the O2/O12 and O3/O7 pairs.

**Figure 11:**
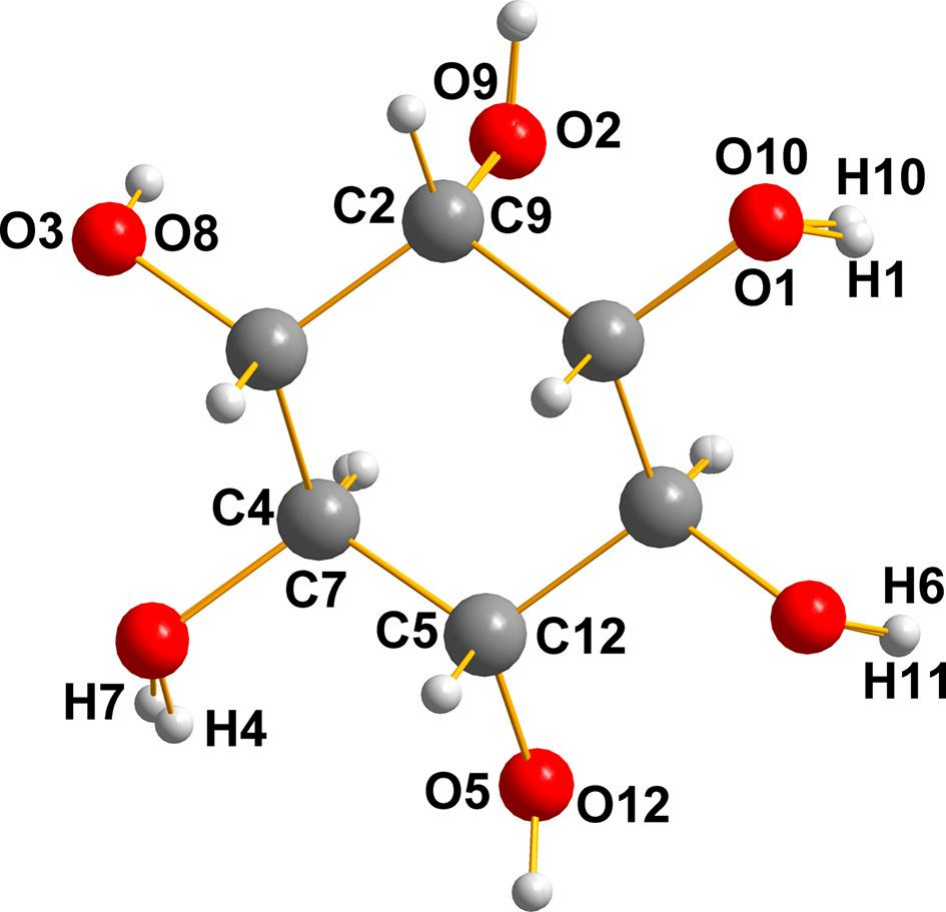
Overlay diagram of the two mol­ecules of inositol (II).

It is notable that the heavy-atom skeleton (C and O) matches so exactly that one can hardly discern the fact that there are two independent mol­ecules superimposed on one another here. The most notable differences are in the cases of H4 with H7 and H1 with H10. A list of the O—H⋯O hydrogen-bond distances for (II) is given in Table 3 ▸. This superposition diagram (overlay, Fig. 11 ▸) was generated by *Mercury* (Groom *et al.*, 2016 ▸) and *DIAMOND* (Putz & Brandenburg, 2019 ▸).

Inositol is not chiral, which may appear as odd at first, since we are accustomed to the fact that the most common sugars we deal with are so. However, this is a misconception inasmuch as there are many nonchiral sugars, as can be found in standard sources. Myo-inositol is an inter­esting sugar present in the brain, as well as other tissues, where it mediates cell transduction in response to certain hormones and neurotransmitters. It is active in processes such as growth and osmoregulation. Thus, it was of more than passing inter­est in finding it present in a confiscated packet of street drugs where it was obviously being used as a diluent to maximize profits of the dealers. Usually, the diluents are powdered milk or other common materials, not a more sophisticated material such as myo-isositol.

Space group and unit-cell constant determination (see Table 2 ▸) revealed that these crystals are monoclinic (space group *P*2_1_/*n*) and whose unit-cell constants exactly matched those of CSD refcode MYINOL02 (Rebecca *et al.*, 2012 ▸); these two are identical having been determined at 100 K and refined to basically the same *R* factors. But, a most important issue is that ours is the only documented sample of a street drug diluent obtained from a police seizure, whereas MYINOL02 was obtained from an extract of Asian Dragon Fruit. Thus, the current structure must be characterized in its totality in order to be used as a reference standard for future legal proceedings.

**Figure 12:**
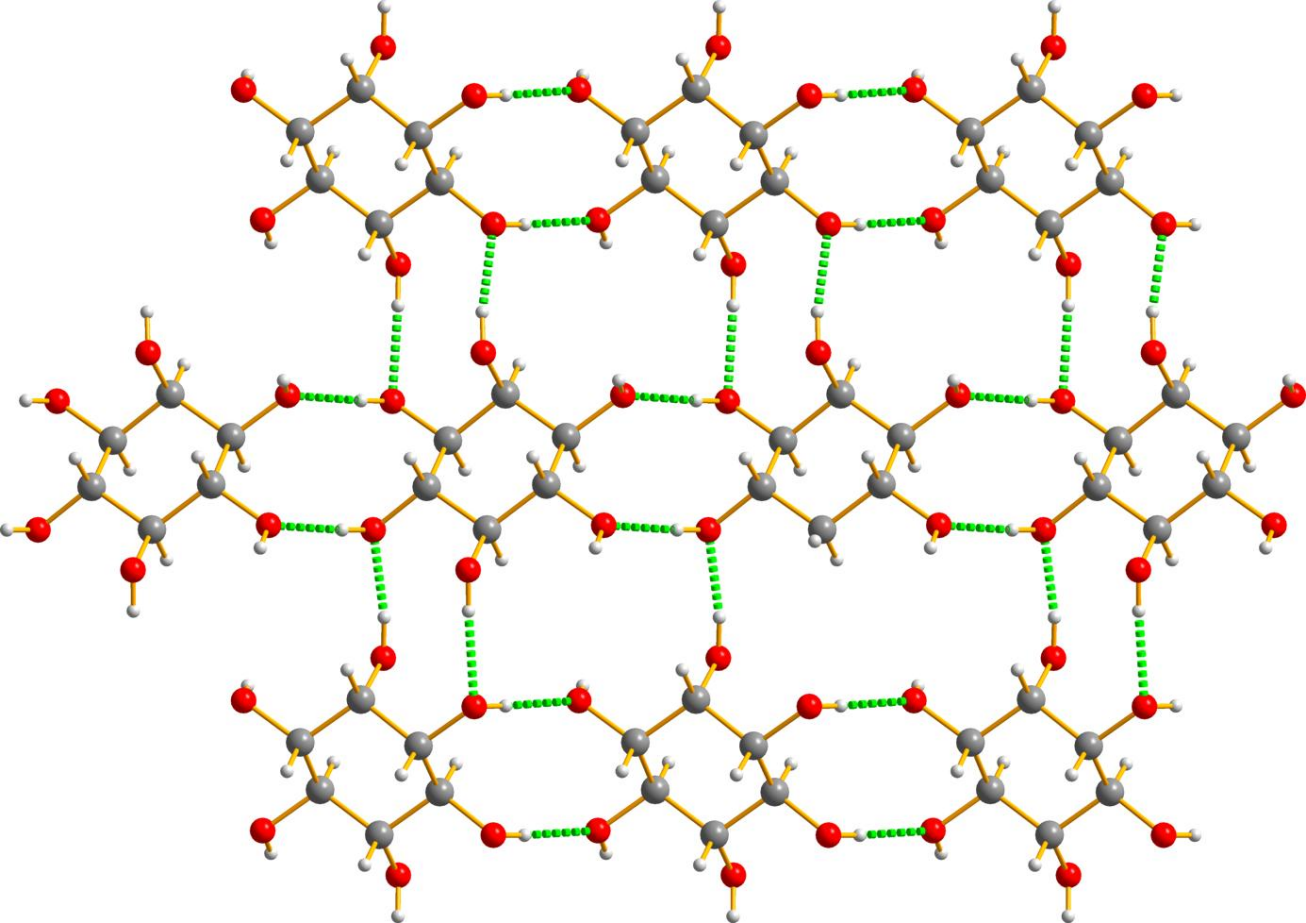
The intricate hydrogen bonding in (II) is quite powerful. The numerical data are presented in Table 3 ▸.

These sugar mol­ecules are linked by a very elaborate set of three-dimensional hydrogen bonds that are illustrated in Fig. 12 ▸.

## Discussion

### Gas chromatography

In the GC–EI–MS analysis of the drug com­pound α-D2PV, (I), an additional peak was observed at retention time 11.20 min, 0.17 min after the com­pound of inter­est at 11.03 min. This peak is routinely observed in cathinone samples due to thermal degradation occurring in the injection port. In a study of 18 cathinones, including pyrrolidino examples, Kerrigan *et al.* (2016 ▸) described the oxidative degradation causing the loss of two H atoms, yielding the 2 Da mass shift in both the mol­ecular ion and the base peak that was observed in the mass spectrum of the additional peak in this com­pound.

### π–π bonding between the drug mol­ecules

The criterion for meaningful contacts between aromatic fragments labeled ‘π–π inter­actions’ in the report by Janiak (2000 ▸) suggests that, given the experimental data available (see Figs. 7 ▸ and 8 ▸, and relevant commentary therein), the range of 3.3–3.8 Å is very reasonable indeed. Using that as an acceptable gauge, our cationic drug mol­ecules have powerful π–π inter­actions, which play a very obvious role in stabilizing the lattice herein described, being 3.6600 (17)–3.6985 (17) Å for all six carbon pairs.

## Conclusions

We were fortunate to obtain a confiscated packet of street drugs con­tain­ing both the opiate and its diluent. They were com­pletely characterized by a variety of analytical methods described above, including a full structural determination of both of its crystalline contents since, helpfully, both were present as high-quality X-ray analysis specimens.

## Supplementary Material

Crystal structure: contains datablock(s) I, II, global. DOI: 10.1107/S2053229624000561/eq3015sup1.cif

Structure factors: contains datablock(s) I. DOI: 10.1107/S2053229624000561/eq3015Isup2.hkl

Structure factors: contains datablock(s) II. DOI: 10.1107/S2053229624000561/eq3015IIsup3.hkl

Supporting information file. DOI: 10.1107/S2053229624000561/eq3015Isup4.cml

Supporting information file. DOI: 10.1107/S2053229624000561/eq3015IIsup5.cml

Additional figures. DOI: 10.1107/S2053229624000561/eq3015sup6.pdf

CCDC references: 2193866, 2193865

## Figures and Tables

**Table 1:** Mass spectrometry parameters for the analysis of α-D2PV, (I)

Instrumental method for seized drug analysis		
Instrument	Thermo Scientific TRACE 1310 GC – ISQ-LT	
Injection mode	splitless	splitless time 1.0 min
GC column	Restek RTX-5Sil MS, 30 m × 0.25 mm × 0.25 µm	
Carrier gas He (99.999%)	Flow 1.0 ml min^−1^, constant flow	
Injector temperature:	220 °C	
Temperature program	65 °C, 2 min	
	30 °C min^−1^ to 150 °C	
	30 °C min^−1^ to 300 °C	
	10 min hold	
Transfer line temperature	280 °C	
Total analysis time	22.83 min	
TriPlus RSH autosampler	Injection volume 1 µl	
ISQ-LT MS ionization mode EI	70 eV	
Ion source temperature	200 °C	
Full scan	45–500 *m*/*z*	

**Table 2:** Experimental details Experiments were carried out at 100 K with Cu *K*α radiation using a Rigaku XtaLAB Synergy Dualflex diffractometer with a HyPix detector. H atoms were treated by a mixture of independent and constrained refinement.

	(I)	(II)
Crystal data		
Chemical formula	2C_18_H_20_NO^+^·2Cl^−^·H_2_O	C_6_H_12_O_6_
*M*_r_	621.62	180.16
Crystal system, space group	Monoclinic, *C*2/*c*	Monoclinic, *P*2_1_/*n*
*a*, *b*, *c* (Å)	13.8926 (1), 11.9663 (1), 19.3872 (1)	6.61708 (6), 12.0474 (1), 18.88721 (19)
β (°)	100.384 (1)	93.9791 (8)
*V* (Å^3^)	3170.20 (4)	1502.04 (2)
*Z*	4	8
μ (mm^−1^)	2.15	1.26
Crystal size (mm)	0.27 × 0.20 × 0.11	0.22 × 0.10 × 0.08

Data collection		
Absorption correction	Multi-scan (*CrysAlis PRO*; Rigaku OD, 2022 ▸)	Gaussian (*CrysAlis PRO*; Rigaku OD, 2022 ▸)
*T*_min_, *T*_max_	0.808, 1.000	0.607, 1.000
No. of measured, independent and observed [*I* > 2σ(*I*)] reflections	58892, 3260, 3089	55215, 3168, 2697
*R*_int_	0.043	0.064
(sin θ/λ)_max_ (Å^−1^)	0.630	0.631

Refinement		
*R*[*F* ^2^ > 2σ(*F* ^2^)], *wR*(*F* ^2^), *S*	0.031, 0.082, 1.09	0.038, 0.110, 1.06
No. of reflections	3260	3168
No. of parameters	203	253
Δρ_max_, Δρ_min_ (e Å^−3^)	0.26, −0.21	0.25, −0.26

Computer programs: *CrysAlis PRO* (Rigaku OD, 2022 ▸), *SHELXT2018* (Sheldrick, 2015*a* ▸), *SHELXL2018* (Sheldrick, 2015*b* ▸), *Mercury* (Macrae *et al.*, 2020 ▸) and *DIAMOND* (Putz & Brandenburg, 2019 ▸).

**Table 3:** Hydrogen-bond geometry (Å, °) for (II)

*D*—H⋯*A*	*D*—H	H⋯*A*	*D*⋯*A*	*D*—H⋯*A*
O1—H1⋯O3^i^	0.831 (19)	1.85 (2)	2.6771 (14)	175.1 (17)
O2—H2⋯O6^ii^	0.854 (19)	1.779 (19)	2.6274 (13)	171.4 (17)
O3—H3⋯O12	0.853 (19)	1.885 (19)	2.7229 (14)	167.3 (17)
O4—H4⋯O10^iii^	0.842 (19)	2.071 (19)	2.8461 (14)	152.9 (16)
O5—H5⋯O1^iv^	0.859 (19)	1.922 (19)	2.7797 (13)	176.8 (17)
O6—H6⋯O4^i^	0.852 (19)	1.79 (2)	2.6403 (14)	171.8 (17)
O7—H7⋯O2	0.854 (19)	1.861 (19)	2.6915 (14)	163.8 (16)
O8—H8⋯O1^ii^	0.869 (19)	1.979 (19)	2.7943 (14)	155.6 (16)
O9—H9⋯O10^v^	0.850 (19)	1.934 (19)	2.7767 (14)	171.3 (17)
O10—H10⋯O8^vi^	0.871 (19)	1.865 (19)	2.7228 (14)	167.5 (16)
O11—H11⋯O7^vi^	0.804 (19)	1.838 (19)	2.6382 (14)	173.8 (18)
O12—H12⋯O11^iii^	0.829 (19)	1.843 (19)	2.6671 (14)	172.3 (17)

Symmetry codes: (i) 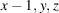 ; (ii) 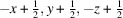 ; (iii) 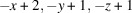 ; (iv) 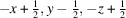 ; (v) 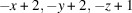 ; (vi) 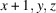 .
