# Supplementary material for: Crystal structure and analytical profile of 1,2-di­phenyl-2-pyrrolidin-1-yl­ethanone hydro­chloride or ‘α-D2PV’: a synthetic cathinone seized by law enforcement, along with its diluent sugar, myo-inositol
Source: Acta Crystallogr C Struct Chem. 2024 Jan 22;80(Pt 4):91–7. doi: 10.1107/S2053229624000561 (PMC10996188; doi:10.1107/S2053229624000561)

STRUCTURAL CHEMISTRY

Volume 80 (2024)

Supporting information for article:

Crystal structure and analytical profile of 1,2-diphenyl-2-pyrrolidin-1-ylethanone hydrochloride or `α-D2PV': a synthetic cathinone seized by law enforcement, along with its diluent sugar, myo-inositol

Matthew R. Wood, Ivan Bernal and Roger A. Lalancette

ORTEP diagram for (**I**). Ellipsoids are plotted at the 40% level.

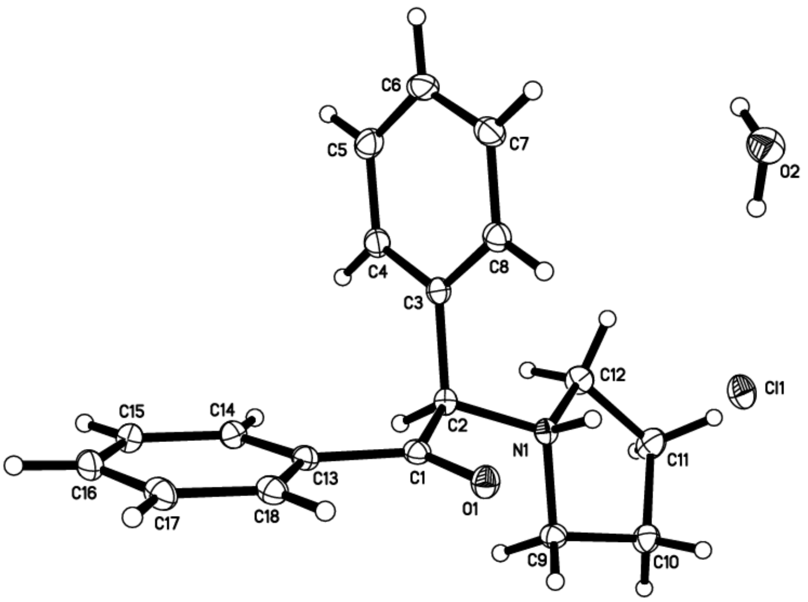

ORTEP diagram for (**II**). Ellipsoids are plotted at the 40% level.

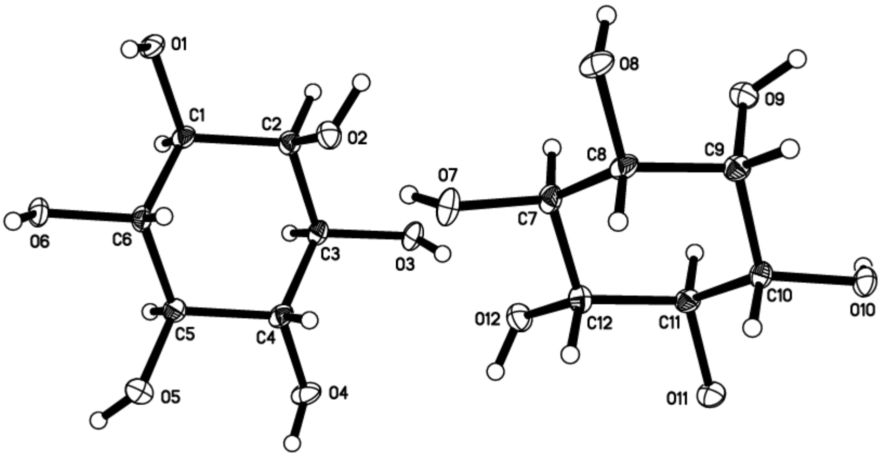

Supplement: Supplementary file 6 [file c-80-00091-sup6.pdf]
